# Unveiling the adaptive strategies in *Blumea Balsamifera* roots under salt stress through integrated physiological and multi-omics analysis

**DOI:** 10.3389/fpls.2026.1970547

**Published:** 2026-10-07

**Authors:** Zejun Mo, Changmao Guo, Su Chen, Kailang Mu, Zhigang Ju, Yuchen Liu, Yuan Yuan, Yuxin Pang

**Affiliations:** 1 College of Pharmaceutical Sciences, Guizhou University of Traditional Chinese Medicine, Guiyang, China; 2 Guizhou Key Laboratory for Germplasm Innovation and Resource-Efficient Utilization of Dao-di Herbs, Guiyang, China; 3 College of Biosciences and Biopharmaceutics, Guangdong Pharmaceutical University, Guangzhou, China

**Keywords:** adaptive mechanisms, *Blumea balsamifera*, multi-omics analysis, root, salt stress

## Abstract

**Background:**

Soil salinization is a growing constraint on agricultural productivity, yet the adaptive mechanisms of many medicinal plants—particularly *Blumea balsamifera*, a species of significant economic and pharmacological value—remain poorly understood. In this study, five treatments—CK (control), LS (low salt), MS (medium salt), HS (high salt), and EHS (extremely high salt)—were established to systematically investigate the comprehensive effects of salt stress on the growth, physiological metabolism, and molecular regulatory networks of *B. balsamifera.*

**Results:**

The results showed that salt stress significantly inhibited the growth of *B. balsamifera*, causing leaf chlorosis and wilting. In roots, the activities of antioxidant enzymes (SOD, POD, CAT) and the level of malondialdehyde (MDA) initially increased and then decreased, while the accumulation of osmoregulatory substances (PRO, SS, SP) and lignin (LIG) progressively increased. Transcriptomic analysis identified 5,701 differentially expressed genes (DEGs) shared between the HS vs CK and EHS vs CK comparisons, which were significantly enriched in functional terms including transcription factor activity, cell wall organization, plant hormone signal transduction. Metabolomic analysis identified 590 and 647 differentially accumulated metabolites (DAMs) in the HS vs CK and EHS vs CK comparisons, respectively. Under moderate stress, flavonoid synthesis and cutin/suberin biosynthesis pathways were significantly enriched, whereas under extreme stress, the phenylpropanoid pathway, carbohydrate metabolism pathway, and oxylipin/jasmonate-related metabolism pathways were significantly activated. Integrated multi-omics analysis further revealed that cutin, suberin, and wax biosynthesis, phenylpropanoid biosynthesis, and oxylipin/jasmonate-related metabolism are the core synergistic pathways in the root response of *B. balsamifera* to salt stress, mediating physical barrier construction, chemical defense and structural reinforcement, and lipid signal transduction, respectively.

**Conclusion:**

Collectively, these findings uncover a multilayered adaptive strategy in *B. balsamifera* roots under salt stress, namely physical barrier formation, chemical-structural defense, and membrane lipid signaling. These results provide theoretical insights and key molecular targets for breeding salt-tolerant varieties and developing stress-resilient cultivation strategies.

## Background

Global soil salinization has become a major threat to agricultural sustainability and food security (FAO, 2024). Particularly in coastal, arid, and semi-arid regions, salt stress has emerged as a critical abiotic factor limiting crop growth and reducing yield and quality (Yang and Y.C., 2023). In saline environments, plants face the effects of multiple stresses, including osmotic stress, ionic toxicity, nutrient imbalance, and oxidative damage (Balasubramaniam et al., 2023). In soybean, salt stress significantly reduces root number and length, limiting water and nutrient uptake (El-Esawi et al., 2018); in wheat, total root length, surface area, and lateral root number decrease with increasing salinity (Liu et al., 2024). Photosynthesis is particularly sensitive to salt stress, primarily manifesting as declines in net photosynthetic rate (Pn), stomatal conductance (Gs), and transpiration rate (Tr) (Munns and Tester, 2008). Salt stress imposes multiple detrimental effects on photosynthetic machinery across different plant species. In response to salt stress, plants exhibit deep adaptive remodeling across growth and development, physiological metabolism, and molecular regulatory networks (Yang and Guo, 2018). Thus, a comprehensive understanding of the physiological and molecular mechanisms underlying plant salt tolerance is crucial for breeding salt-resistant crop varieties and for the sustainable utilization of saline-alkali land resources.

The effects of salt stress on plants are multifaceted (Kantilal Chande, 2020; Zahra et al., 2022). At the physiological and biochemical level, the root—as the primary organ responsible for sensing soil salinity and mediating ion uptake and selective transport—maintains basic functionality and structural integrity through coordinated regulation of the reactive oxygen species (ROS) metabolic system, osmoregulatory substance accumulation, phytohormone balance, and cell wall properties (Muhammad Adnan Shahid, 2020; Sanwal et al., 2022). Beneath these physiological and biochemical responses lie more intricate processes of metabolic and transcriptional reprogramming. Such coordinated changes in transcription and metabolism may constitute crucial adaptive strategies for plants to cope with environmental stress (Zhao et al., 2021; Li et al., 2024). At the metabolic level, salt stress triggers extensive reprogramming of primary metabolism—including carbohydrate, amino acid, and organic acid pathways—as well as secondary metabolism, such as phenylpropanoid, terpenoid, and alkaloid biosynthesis. These metabolic adjustments enable the reallocation of carbon and nitrogen resources and the production of protective metabolites (Zhu et al., 2022; Al-Khayri et al., 2023). At the transcriptional level, salt stress triggers widespread changes in gene expression. Critical functional genes, including transcription factors, protein kinases, and ion transporters, are coordinately up- or down-regulated, collectively forming a sophisticated regulatory network that orchestrates systemic adaptive responses (Singh and Roychoudhury, 2021; Aizaz et al., 2024). Therefore, integrated multi-omics approaches are essential for deciphering the coordinated metabolic and transcriptional reprogramming underlying plant salt tolerance.

*Blumea balsamifera* (L.) DC., a perennial herbaceous plant of the genus Blumea in the Asteraceae family, is an important characteristic medicinal plant in southern China and a traditional herbal medicine among the Miao, Zhuang, Li, and other ethnic minorities (Ling-Liang Guan, 2012). Its leaves are rich in volatile oils, flavonoids, and terpenoids, which exhibit antibacterial, anti-inflammatory, and antioxidant activities, with broad application prospects in pharmaceutical, spice, and cosmetic industries (Pang et al., 2014; Silalahi, 2021). Currently, research on the abiotic stress biology of *B. balsamifera* has primarily focused on drought and cadmium (Cd) stress. For instance, Guo et al. found that drought significantly inhibited the growth and development of *B. balsamifera*, and identified 18 key drought-responsive genes, including *CAB6*, *40S ribosomal protein S7*, and *S25-4 (*Guo et al., 2025). Chen et al. preliminarily elucidated the molecular response mechanisms of *B. balsamifera* to Cd stress at the transcriptomic level (Chen Zihan, 2022). However, despite the increasing severity of global soil salinization, research on the salt stress response of *B. balsamifera* remains scarce. Guo et al. (2026) established five salt concentration treatments and performed integrated multi-omics analysis on *B. balsamifera* leaves, demonstrating that oxidative phosphorylation, flavone and flavonol biosynthesis, and cutin, suberine, and wax biosynthesis were the core response pathways. Notably, this previous work focused exclusively on leaf tissue; the root—the primary organ for salinity perception and ion regulation—remains largely unexplored.

Recent advances in high-throughput sequencing and metabolomics technologies have provided powerful tools for plant stress biology research, enabling the dissection of molecular mechanisms underlying plant responses to abiotic stress at the systems level (Tao Yu, 2025). Under salt stress conditions, plants often reallocate carbon resources from primary to secondary metabolism, enhancing the production of flavonoids, terpenoids, and phenylpropanoids—compounds that serve both as antioxidants and as structural defenders (MuhammadTanveer Akhtar, 2026). For medicinal plants such as *B. balsamifera*, whose pharmacological value depends on the accumulation of bioactive secondary metabolites, understanding how salt stress modulates these metabolic pathways is of particular importance. However, the specific effects of salt stress on secondary metabolism in *B. balsamifera*—particularly at the root level—remain unexplored.

Therefore, this study focuses on *B. balsamifera*, with particular emphasis on its root, aiming to comprehensively elucidate the salt stress response mechanisms of this species through systematic experimental design and integrated multi-omics approaches. The findings are expected to provide theoretical foundations and genetic resources for molecular marker-assisted breeding of salt-tolerant *B. balsamifera* and stress-resilient cultivation management, while also serving as a valuable reference for studies on salt tolerance mechanisms in other medicinal plants or non-model plant species.

## Results

### Effects of salt stress on phenotype in *B. balsamifera*

With respect to phenotypic characteristics, *B. balsamifera* displayed evident responses to salt stress following 24 days of treatment with solutions of different salt concentrations (**Figure 1**). Under normal growth conditions (CK), the plant leaves are stretched, with a bright green color and vigorous growth. With increase of salt concentration, the color of plant leaves becomes lighter, the edges show mild wilting, and the leaf extension decreases. In the HS treatment group, yellowing or local withered spots were observed on the leaves, and the overall biomass accumulation was significantly inhibited. It can be seen that salt stress has a significant negative impact on the morphogenesis and growth development of *B. balsamifera*.

**Figure 1:**
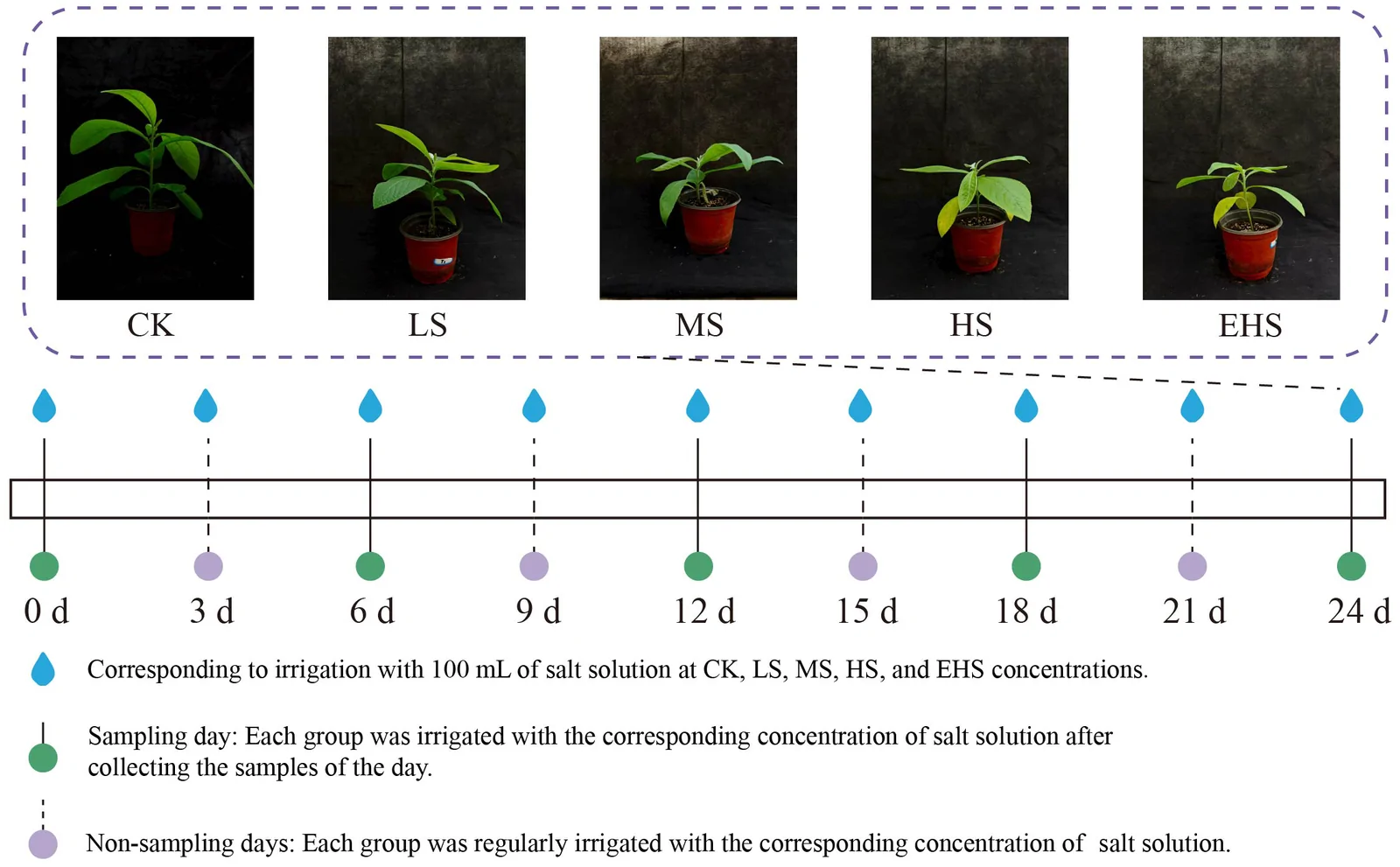
The effect of salt stress on the phenotype of *B. balsamifera*. Phenotypic characteristics of each treatment group on day 24. CK, control (0 mmol·L^−^¹ NaCl); LS, low salt (50 mmol·L^−^¹); MS, medium salt (100 mmol·L^−^¹); HS, high salt (150 mmol·L^−^¹); EHS, extremely high salt (200 mmol·L^−^¹). The sampling timeline is shown below, with green dots indicating sampling days (days 0, 6, 12, 18, 24) and purple dots indicating non-sampling irrigation days.

### Effects of salt stress on physiological indices of the roots of *B. balsamifera*

The root is the primary organ that first perceives and responds to soil salinity. To investigate the effects of salt stress on the physiological and biochemical metabolism of *B. balsamifera* roots, key indicators such as ROS scavenging system, osmoregulatory substances, and lignin content were measured. The results showed that salt stress significantly altered the physiological response pattern of *B. balsamifera* roots. Under LS, the activities of SOD, POD, and CAT showed an induced increase trend in the early stages of stress. However, under MS to EHS, with prolonged stress time, the activities of SOD and POD showed a significant decrease after reaching their peak (*P* < 0.05), while CAT activity was continuously inhibited under HS and above stress (**Figures 2B–D**). Correspondingly, the content of MDA, a product of membrane lipid peroxidation, reached its peak at 12 days after treatment, indicating that severe oxidative damage had occurred (**Figure 2A**). In terms of osmoregulatory substances (**Figures 2F, G**), the content of PRO and SS significantly increased under salt stress, and showed a clear concentration- and time-dependent patterns. The SP content initially increased and then decreased (**Figure 2H**). In addition, the LIG content generally showed an increasing trend under salt stress, with significant accumulation particularly in the middle and late stages of MS and HS treatments (**Figure 2E**).

**Figure 2:**
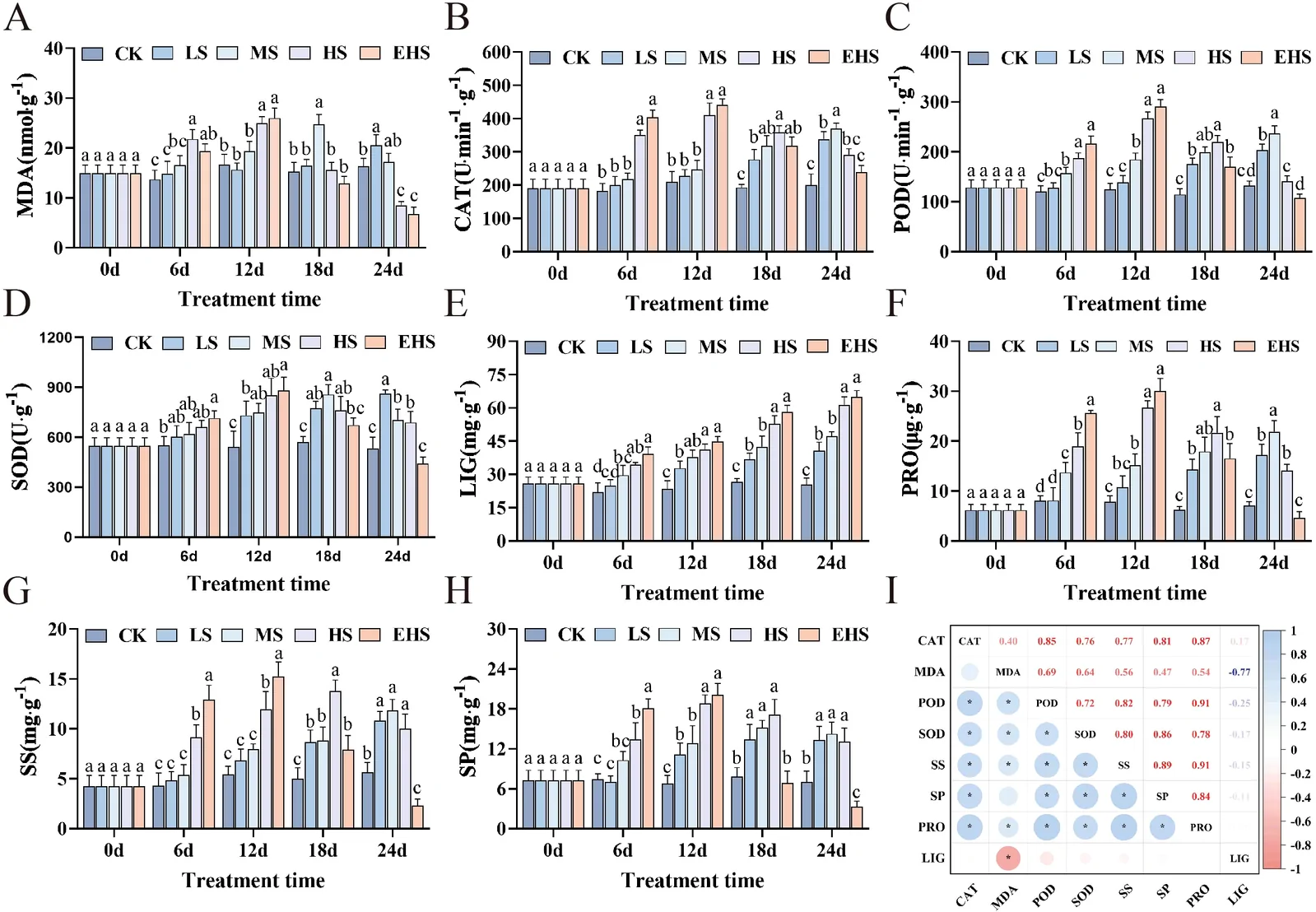
The effect of salt stress on physiological and biochemical indicators of the root of *B. balsamifera*. **(A)** Malondialdehyde (MDA) content. **(B)** Catalase (CAT) activity. **(C)** Peroxidase (POD) activity. **(D)** Superoxide dismutase (SOD) activity. **(E)** Lignin (LIG) content. **(F)** Proline (PRO) content. **(G)** Soluble sugar (SS) content. **(H)** Soluble protein (SP) content. Data are presented as mean ± SD (n = 3 biological replicates). Different lowercase letters indicate significant differences among treatments at the same time point (Duncan’s new multiple range test, *P* ≤ 0.05). **(I)** Heatmap of pearson correlation coefficients among physiological indices; asterisks indicate significant correlations (*P* ≤ 0.05).

Correlation heatmap analysis further revealed the intrinsic relationships among the indicators (**Figure 2I**). MDA content was significantly negatively correlated with SOD and POD activities, but significantly positively correlated with PRO and SS contents, indicating that the inactivation of the antioxidant enzyme system and the accumulation of osmotic regulatory substances were both closely associated with the process of membrane lipid peroxidation. SOD, POD, and CAT activities were significantly positively correlated with one another, suggesting that the three enzymes synergistically participate in the scavenging of ROS.

### Transcriptomic profiling analysis of *B. balsamifera* roots under salt stress

To further reveal the response mechanism of *B. balsamifera* roots under salt stress, transcriptome sequencing analysis was performed in this study. The correlation heatmap between samples showed that the biological replicates within each group are highly correlated, while there are significant transcriptional differences between different treatment groups (HS, EHS, and CK) (**Figure 3A**), confirming the significance and reliability of salt stress treatment and experimental data. Analysis of gene expression levels showed that in the HS vs CK comparison, a total of 10,422 DEGs were identified, among which 5,408 were up-regulated and 5,014 were down-regulated (**Figure 3B**). In the EHS vs CK comparison, the total number of DEGs increased to 13,258, with 7,130 up-regulated and 6,128 down-regulated (**Figure 3C**). A total of 5,701 DEGs were commonly expressed in both comparison groups (**Figures 3D, E**). These DEGs may represent the molecular basis underlying the response of *B. balsamifera* to salt stress.

**Figure 3:**
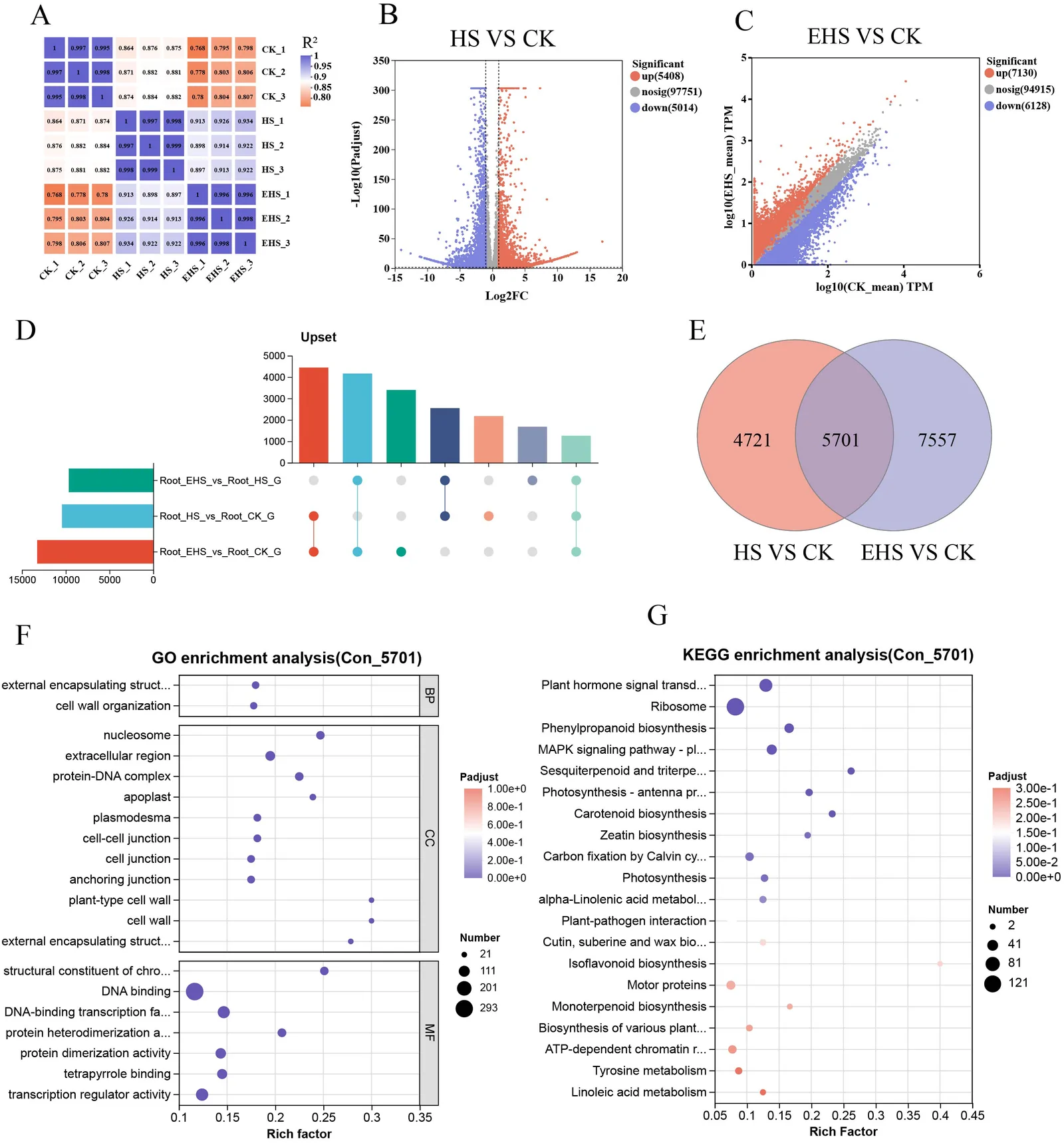
Analysis of Transcriptome expression of *B. balsamifera* roots under salt stress. **(A)** Sample correlation heatmap. **(B, C)** Volcanic diagram of differentially expressed genes (DEGs): showing the distribution of DEGs in HS vs CK and EHS vs CK, respectively. The red dots represent up-regulated DEGs, the purple dots represent down-regulated DEGs, and the gray dots represent no significant DEGs. **(D)** Gene numbers comparison and analysis of the three treatment groups. **(E)** Interactive Venn diagram of DEGs for the two comparison groups. **(F)** GO functional enrichment analysis of 5701 differentially expressed genes. **(G)** KEGG functional annotation analysis of 5701 differentially expressed genes.

GO functional enrichment analysis revealed the main molecular functions and biological processes of the DEGs (**Figure 3F**). The results showed that 5,701 DEGs were significantly enriched in terms such as DNA-binding transcription factor activity, Protein-DNA complex, Transcription regulator activity, and Cell wall organization. KEGG pathway enrichment analysis elucidated the potential functional orientation of transcriptional regulation from the perspective of metabolic and signaling pathways (**Figure 3G**). The results showed that DEGs were significantly enriched in pathways such as Plant hormone signal transduction, Phenylpropanoid biosynthesis, MAPK signaling pathway-plant, and Zeatin biosynthesis.

### Metabolomic profiling analysis of *B. balsamifera* roots under salt stress

To systematically analyze the response mechanism of the metabolic network in *B. balsamifera* roots under salt stress, untargeted metabolomics analysis was performed in this study. The results of principal component analysis (PCA) showed that the metabolite profiles of the CK, HS, and EHS showed a clear separation trend, with biological replicates within each group clustering well, proving the reliability of the experiment and the significant effect of stress treatment (**Figure 4A**). Differential accumulated metabolites (DAMs) analysis revealed that a total of 590 DAMs were identified in HS vs CK, among which 366 were up-regulated and 224 were down-regulated (**Figure 4B**). In EHS vs CK, a total of 647 DAMs were identified, with 443 up-regulated and 204 down-regulated (**Figure 4C**). These results indicated that both HS and EHS triggered substantial metabolic reprogramming, with up-regulated metabolites predominating overall.

**Figure 4:**
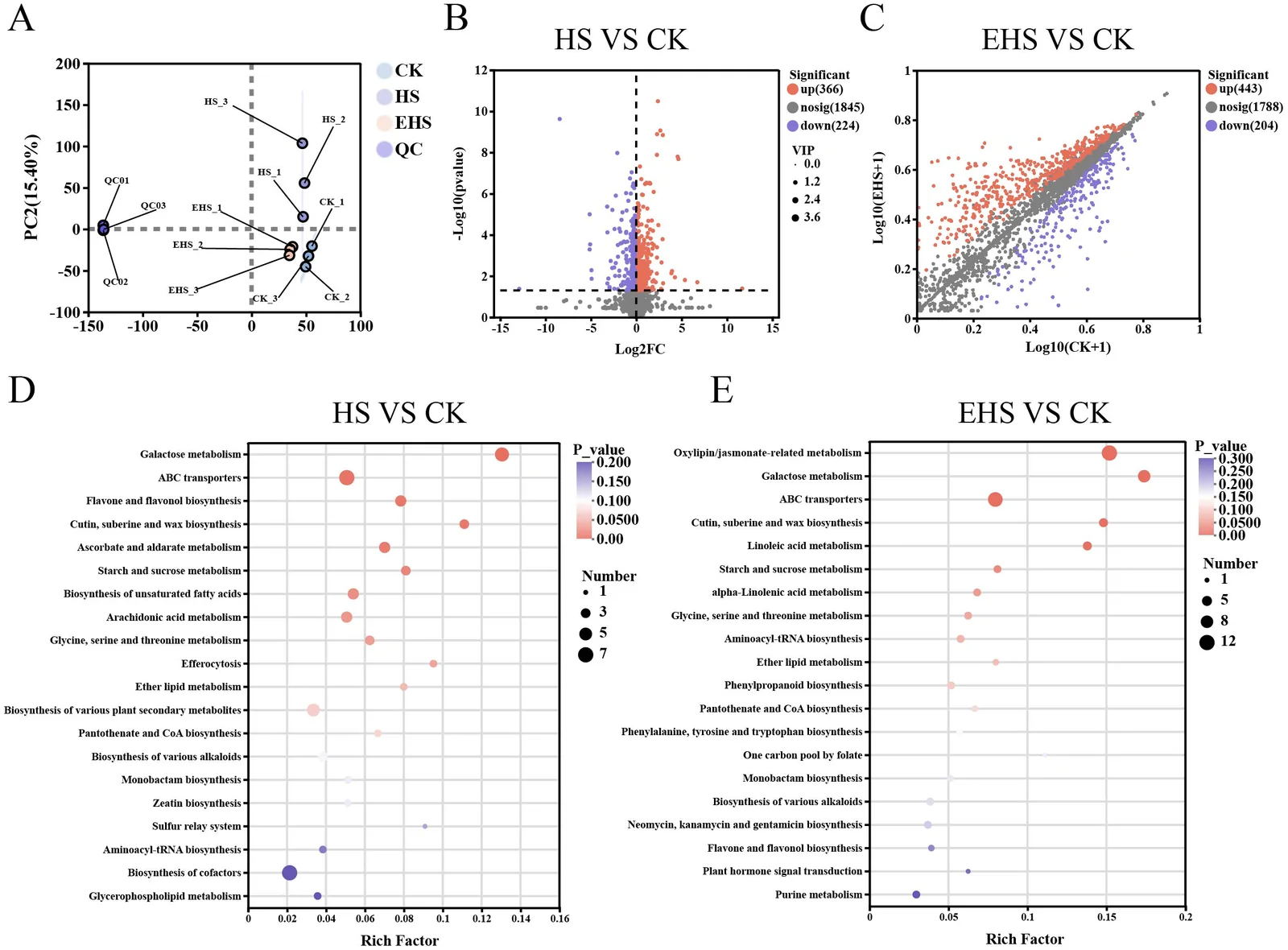
Analysis of metabolic profile expression of *B. balsamifera* roots under salt stress. **(A)** Principal component analysis. Each point represents a repetition in the grouped experiment, and different colors distinguish different groups. **(B, C)** Volcanic diagram of differential metabolites (DAMs): showing the distribution of DAMs in HS vs CK and EHS vs CK, respectively. The red dots represent up-regulated DAMs, the purple dots represent down-regulated DAMs, and the gray dots represent no significant DAMs. **(D, E)** KEGG pathway enrichment analysis. KEGG enrichment of differential metabolites in HS vs CK and EHS vs CK, respectively.

To further elucidate the pathway characteristics of metabolic responses under different intensities of salt stress, KEGG pathway enrichment analysis was performed on the aforementioned DAMs. In the HS vs CK comparison, DAMs were significantly enriched in pathways such as biosynthesis of secondary metabolites, cutin, suberine and wax biosynthesis, ABC transporters, biosynthesis of cofactors, and flavone and flavonol biosynthesis (**Figure 4D**). In contrast, DAMs in the EHS vs CK comparison were significantly enriched in pathways such as galactose metabolism, phenylpropanoid biosynthesis, oxylipin/jasmonate-related metabolism, and starch and sucrose metabolism (**Figure 4E**). These systematic changes at the metabolic level provide important molecular evidence for understanding the adaptive and damage mechanisms of *B. balsamifera* under salt stress.

### Integrated analysis of metabolome and transcriptome

To integrate the metabolic and transcriptional responses of *B. balsamifera* roots under salt stress and elucidate their synergistic regulatory mechanisms, an integrated analysis of dual omics data was performed in this study. Procrustes analysis revealed a significant correlation between metabolite and gene expression profiles across different treatments (**Figure 5A**). By comparing the intersection of KEGG pathway enrichment between DAMs and DEGs in two comparison groups, it was found that in the HS vs CK group, 58 pathways were co-enriched (**Figure 5B**), while in the EHS vs CK group, the number of co-enriched pathways increased to 64 (**Figure 5C**). This result indicates that with increasing stress intensity, metabolite accumulation and gene expression exhibit stronger synergy and consistency at the pathway level. Notably, in both the HS vs CK and EHS vs CK comparison groups, DEGs and DAMs were consistently and significantly co-enriched in pathways such as phenylpropanoid biosynthesis, sesquiterpenoid and triterpenoid biosynthesis, starch and sucrose metabolism, and oxylipin/jasmonate-related metabolism (**Figures 5D, E**). This indicates that regardless of stress intensity, these pathways are continuously activated at both the transcriptional and metabolic levels, constituting the core reprogramming modules of the salt stress response in *B. balsamifera*.

**Figure 5:**
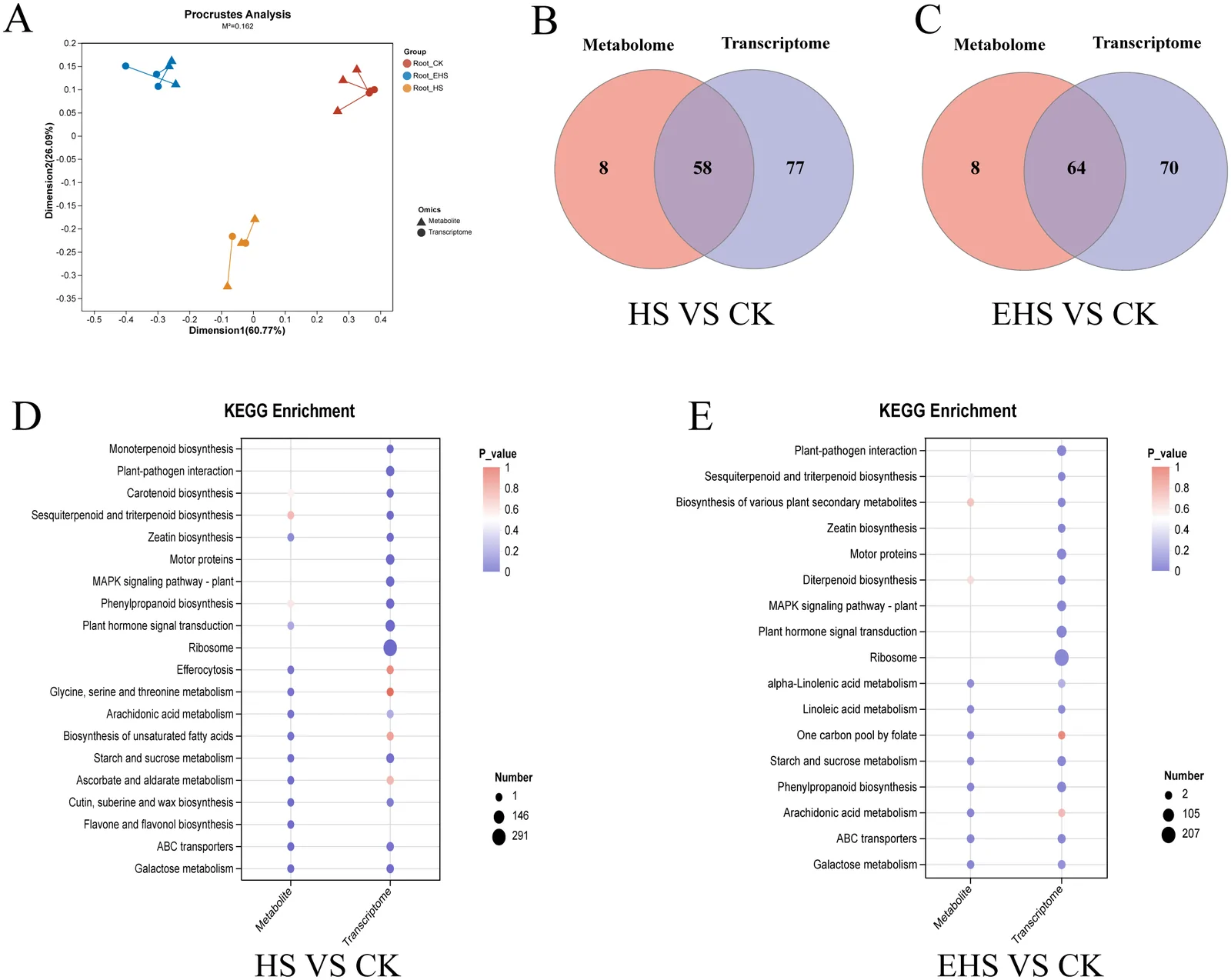
Metabolome and transcriptome combined analysis. **(A)** Procrustes analysis of metabolome and transcriptome results. **(B)** The Venn plot of KEGG annotation results for the metabolome and transcriptome of HS vs CK, with the intersection representing the number of pathways annotated by both sets of data. **(C)** The Venn plot of KEGG annotation results for the metabolome and transcriptome of EHS vs CK, with the intersection representing the number of pathways annotated by both sets of data. **(D)** HS vs CK Metabolome and Transcriptome KEGG Enrichment Pathway Bubble Chart. **(E)** EHS vs CK Metabolome and Transcriptome KEGG Enrichment Pathway Bubble Chart.

### Expression of DAMs and DEGs related to the cutin, suberine, and wax biosynthesis pathways

Based on the integrated analysis of transcriptome and metabolome, *B. balsamifera* exhibits root-specific metabolic and gene expression co-reprogramming in the “Cutin, suberine, and wax biosynthesis” pathway under salt stress. At the metabolic level, several long-chain fatty acid derivatives, serving as key structural monomers of suberin, were significantly accumulated in the roots. Notably, intermediate products such as ω-hydroxy fatty acid (22-hydroxydocosanoic acid) and α,ω-dicarboxylic acid showed continuously increasing contents under salt treatment, indicating that the synthesis of suberin monomers in the root endodermis was specifically activated. Meanwhile, the accumulation of polyhydroxy fatty acids (such as (S)-10,16-dihydroxyhexadecanoic acid) also suggests that suberin-related processes in the roots may be involved in the response to ionic stress. At the gene expression level, consistent with the enhancement of metabolic flow, key genes that catalyze the elongation of fatty acid chains (such as *TRINITYDN25406_c0ug1*) and enzyme genes responsible for fatty acid ω-hydroxylation and oxidation to generate dicarboxylic acids (such as *TRINITYDN69340_c0ug1* and *TRINITYDDN6473_c0ug1*) were generally up-regulated, forming a complete positive regulatory chain from gene transcription to metabolite synthesis (**Figure 6**). The high correlation between the accumulation of metabolites and up-regulation of gene expression at pathway nodes reveals the adaptive strategy of *B. balsamifera* roots to systematically promote cork deposition by precisely driving metabolic flow through transcriptional reprogramming.

**Figure 6:**
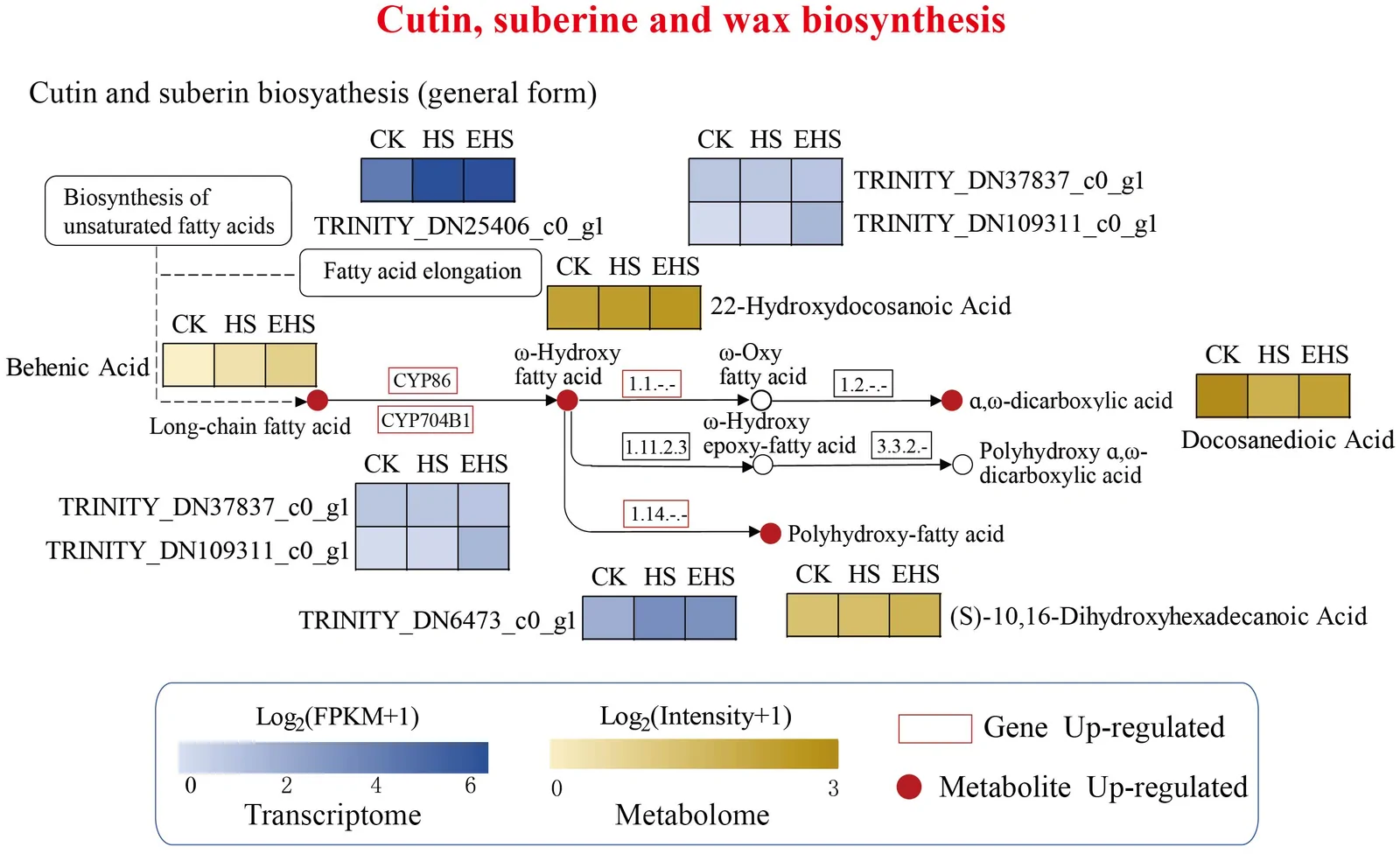
Transcriptome and metabolome integration analysis of the pathways of cuticle, suberin, and wax biosynthesis in *B. balsamifera* under salt stress.

### Expression of DAMs and DEGs related to the phenylpropanoid biosynthesis pathways

Under salt stress, the phenylpropanoid biosynthesis pathway of *B. balsamifera* roots exhibits multi branch synergistic activation of transcription and metabolic remodeling, manifested by significant up-regulation of upstream key genes (such as phenylalanine ammonia lyase genes *TRINITYDN2622C0_g1* and *TRINITYDN42454_c0ug1*), to synchronous accumulation of downstream metabolites (such as lignin precursor 4-Coumaryl Alcohol and P-Coumaroyl CoA) (**Figure 7**). The results showed that most DEGs were continuously up-regulated under salt stress, which was highly consistent with the trend of DAMs, forming a coherent activation pattern from phenylalanine to lignin and flavonoid branches. The differential up-regulation of relevant genes in the pathway, such as 4-coumaroyl CoA ligase (*TRINITY1 DN1660_c0ug1*), cinnamoyl CoA dehydrogenase (*TRINITY1 DN818_c0ug2*), and peroxidase (*TRINITY1 DN78613_c0ug2*, etc.), precisely guides metabolic flow towards different stress-responsive branches, reflecting the preferential allocation of carbon resources to defensive secondary metabolism under salt stress. The response of this pathway is the key molecular basis for the resistance of *B. balsamifera* to salt induced osmotic stress and ion toxicity, providing clear targets for the subsequent excavation of salt resistant key genes and metabolic markers.

**Figure 7:**
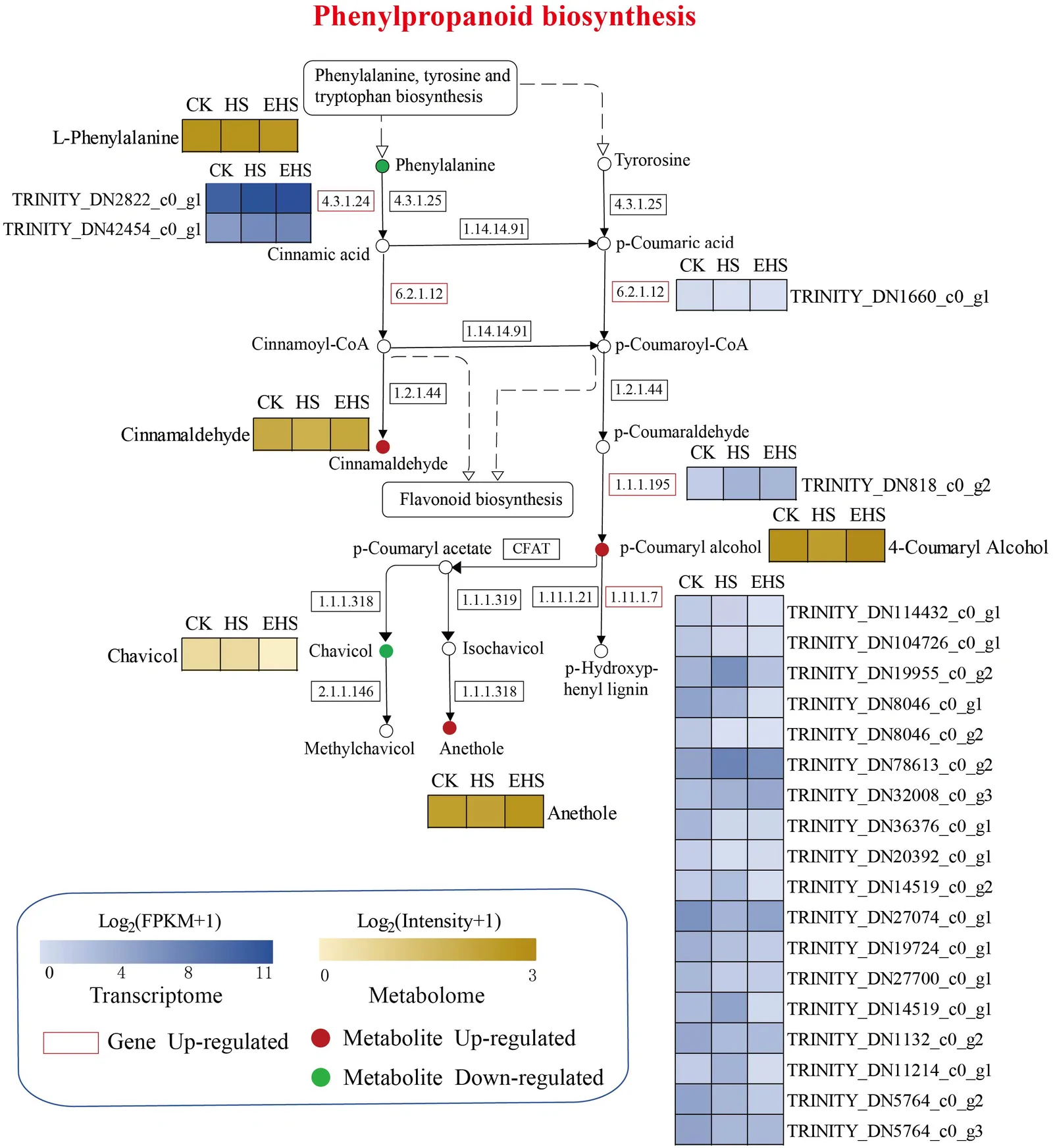
Transcriptome and metabolome integration analysis of the pathways of phenylpropanoid biosynthesis in *B. balsamifera* under salt stress.

### Expression of DAMs and DEGs related to the oxylipin/jasmonate-related metabolism pathways

Based on the integrated analysis of transcriptome and metabolome, the oxylipin/jasmonate-related metabolism pathway in the roots of *B. balsamifera* exhibits significant dynamic reprogramming of metabolism and gene expression under salt stress, indicating that this pathway plays a key role in plant response to salt induced oxidative stress and signal transduction (**Figure 8**). Notably, the prostaglandin-like metabolites annotated in this pathway are likely plant oxylipins or jasmonate-related metabolites rather than true prostaglandins, and this pathway is therefore interpreted as oxylipin/jasmonate-related metabolism. At the metabolic level, multiple oxidized lipid metabolites (including cyclopentenone-containing oxylipins and their derivatives) that exhibited differential accumulation patterns under salt stress. At the gene expression level, significant changes were observed in the expression of multiple key enzyme genes (such as *TRINITYDN981_c0_g1*, *TRINITYDN19710_c0ug1* encoding enzymes that may be involved in oxidized lipid signaling molecules synthesis or modification), with some genes up-regulated under salt stress, consistent with the accumulation trend of downstream metabolites. The expression regulation of these genes may directly affect the synthesis and metabolic flow of oxidized lipid signaling, thereby regulating the adaptive response of roots in salt environments.

**Figure 8:**
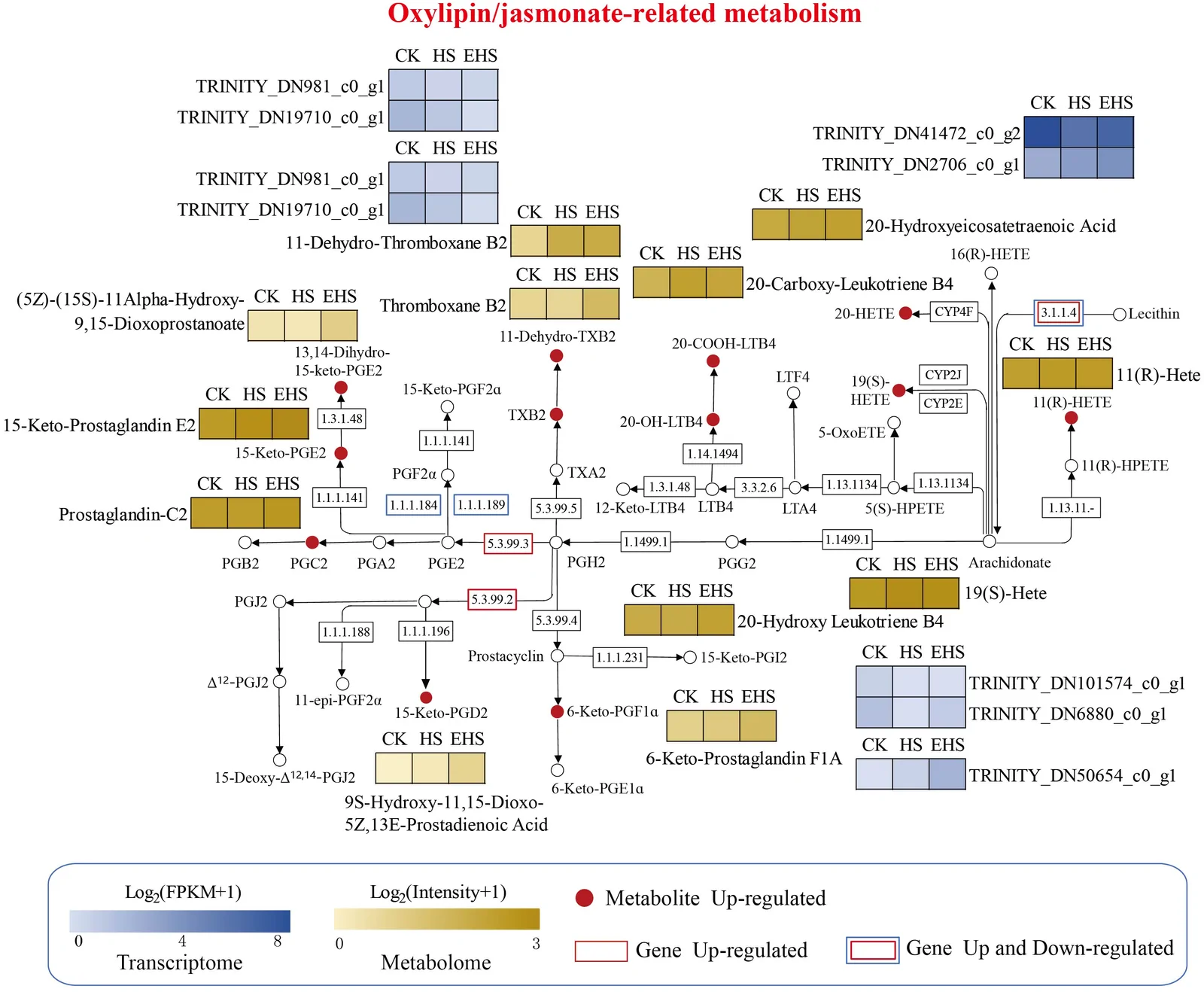
Transcriptome and metabolome integration analysis of the pathways of oxylipin/jasmonate-related metabolism in *B. balsamifera* under salt stress.

### Determination of gene expression levels (RT-qPCR)

To analyze the reliability of transcriptome sequencing results, nine DEGs representing key regulatory nodes in salt stress signaling and oxidative stress responses were selected for RT-qPCR validation. The results showed that the relative expression trends of the selected genes under CK, HS, and EHS treatments were highly consistent with the changes in FPKM values determined by RNA-seq (**Figure 9**). Both exhibited significantly up-regulated or down-regulated expression patterns under salt stress induction, with expression levels further changing as stress intensity increased. This finding experimentally confirmed the accuracy and reproducibility of the transcriptome data. Additionally, the significant differential expression of these genes under stress suggests their potential roles in regulation of salt stress response and adaptation in *B. balsamifera*, providing candidate genes for further functional research. In this study, the RT-qPCR validation set primarily represented signaling and oxidative stress-related genes, and did not include representatives from the cutin/suberin or oxylipin/jasmonate pathways. Future studies with a broader validation set covering all core pathways would further strengthen the conclusions.

**Figure 9:**
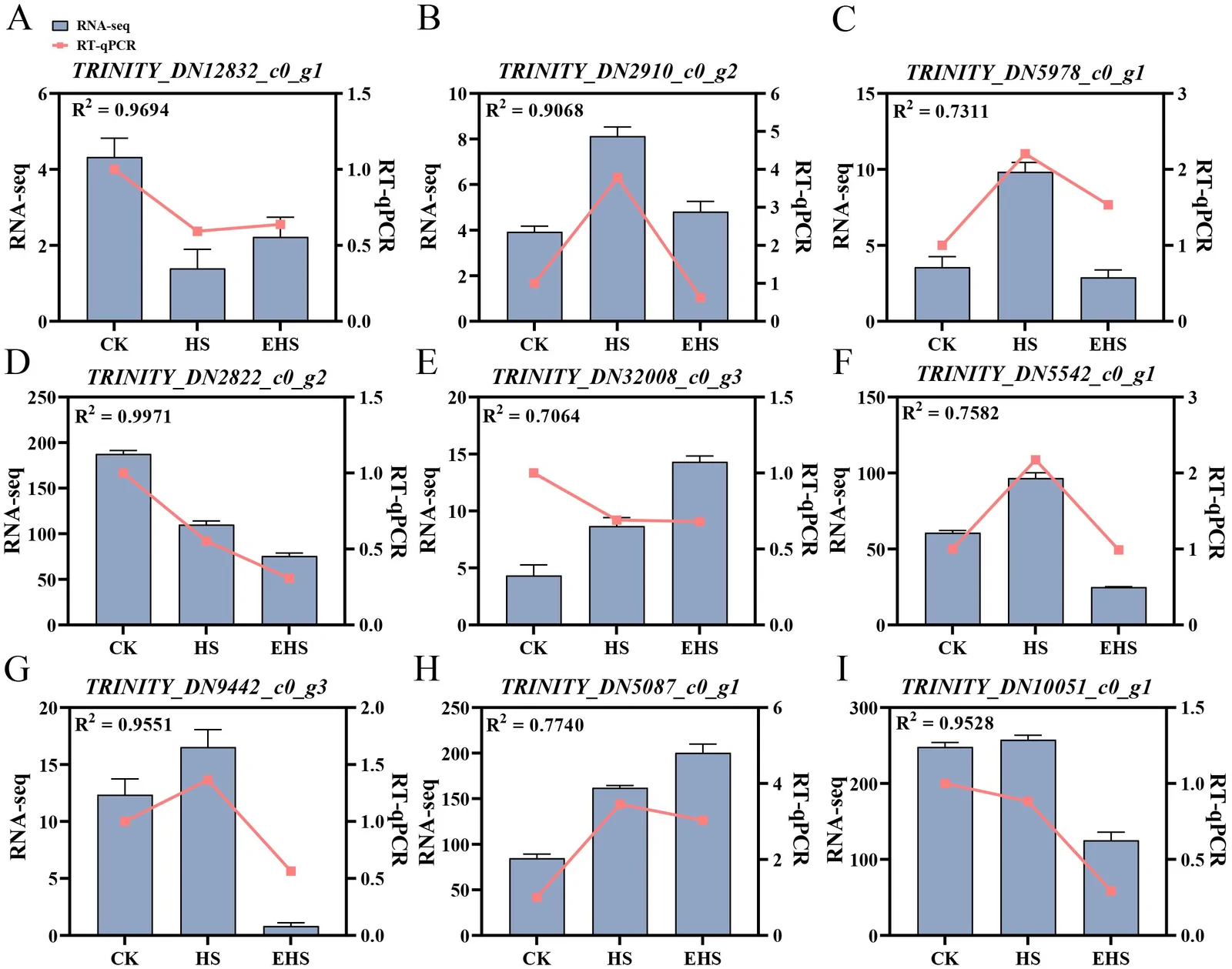
Comparison of gene expression levels of different treatment groups by RNA-seq and RT-qPCR.

Collectively, our multi-omics integration reveals a coordinated, multilayered adaptive strategy in *B. balsamifera* roots under salt stress (**Figure 10**). Three core pathways emerged as synergistically activated: (1) cutin, suberin, and wax biosynthesis, which may contribute to physical barrier reinforcement through the accumulation of hydrophobic polymers (ω-hydroxy fatty acids and α,ω-dicarboxylic acids) and the up-regulation of fatty acid elongases and hydroxylases, thereby restricting apoplastic Na^+^ and water influx; (2) phenylpropanoid biosynthesis, which supports both structural reinforcement via lignin accumulation (validated by increased LIG content and up-regulated PAL, 4CL, and CAD expression) and antioxidant defense via flavonoid synthesis; and (3) oxylipin/jasmonate-related metabolism, which modulates stress signaling and redox homeostasis through the accumulation of oxidized lipid metabolites and the coordinated expression of lipoxygenase-related genes. These pathways do not operate in isolation but form an integrated regulatory network—linking physical barrier formation, chemical-structural defense, and membrane lipid signaling—that collectively underpins root adaptation to salt stress in *B. balsamifera*.

**Figure 10:**
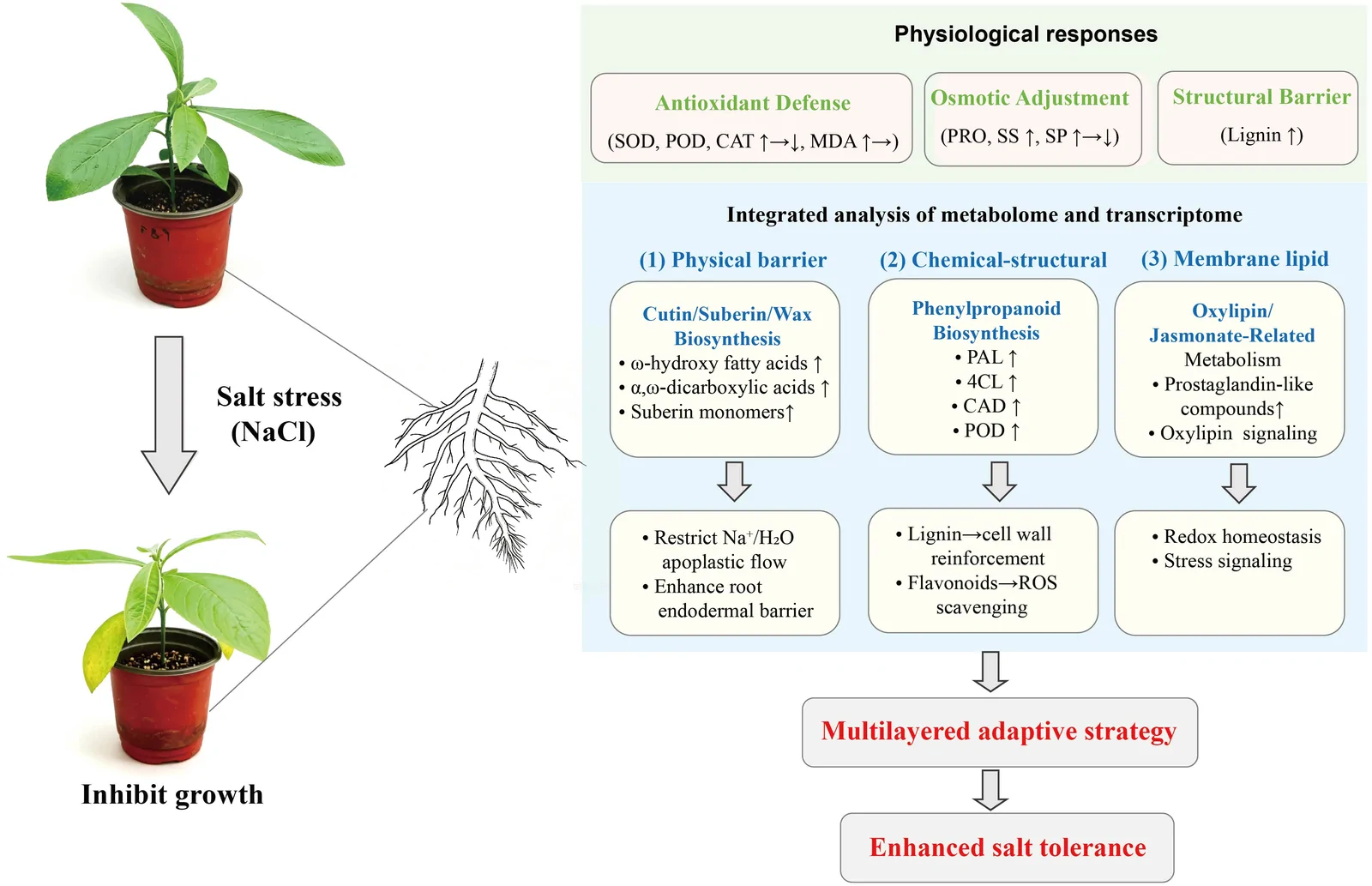
The model of salt stress response mechanisms in *B. balsamifera* roots.

## Discussion

Salt stress, as one of the main abiotic stresses affecting crop yield and ecological environment worldwide, has a complex mechanism of action involving multi-level effects from plant morphological development to molecular regulatory networks (Zhou et al., 2024; Tibesigwa et al., 2025). This study focuses on the characteristic medicinal plant *B. balsamifera* and explores the systematic effects of salt stress on its phenotype, photosynthetic physiology, root physiological characteristics, as well as transcription and metabolism. The research results reveal the complex adaptive mechanisms and potential damage processes of *B. balsamifera* in response to salt stress at the biological level, and provide comprehensive data support and theoretical basis for understanding its salt tolerance regulatory network.

Salt stress significantly inhibits the growth of *B. balsamifera*, causing leaf wilting, yellowing, local necrosis, and reduced biomass, consistent with our companion study on the global physiological responses of this species (Guo et al., 2026). These phenotypic manifestations represent a comprehensive response to osmotic stress, ion toxicity, and secondary oxidative damage (Munns and Tester, 2008; Yang and Guo, 2018). The root, as the first line of defense for directly sensing changes in soil salinity, plays a critical role in salt stress responses. Although leaves and stems are also involved in the overall response, it is the physiological and biochemical responses of the root that are key to determining the plant’s overall salt tolerance (Cui et al., 2025; Tariq et al., 2026).

This study found that MS stress induced increases in the activities of SOD, POD, and CAT, which is a typical defense response of plants to clear salt induced ROS and protect cell membrane structure from oxidative damage (Li et al., 2020). However, the oxidative stress generated by high-intensity or prolonged salt stress has exceeded the buffering capacity of the root enzyme clearance system, leading to irreversible accumulation of oxidative damage. Parallel to oxidative defense are the strategies of osmotic adjustment and structural stabilization. The stress dependent significant accumulation of PRO and SS content is a common pattern for plants to maintain cell osmotic potential, stabilize macromolecular structures, and protect organelles by synthesizing compatible solutes (Slama et al., 2015). In this study, lignin content showed a significant positive correlation with both stress intensity and MDA content during the later stages of stress. This may reflect a trade-off: enhanced lignification likely represents an adaptive barrier-reinforcement mechanism, whereas excessive lignification—and its associated metabolic costs—under severe stress may be linked to progressive cellular damage (Swetha et al., 2024; Bawa et al., 2026). It should be noted that the time × treatment interaction was not formally tested in this study. Future work using a full two-factor design with interaction terms would help further resolve the temporal dynamics of salt responses in *B. balsamifera* roots.

Beyond these physiological adjustments, the underlying molecular reprogramming provides deeper insight into the adaptive capacity of *B. balsamifera* roots. Untargeted metabolomics analysis revealed the dynamic response of the metabolic network in the roots of *B. balsamifera* to salt stress. This study found that under HS stress, the metabolic response was prominently enhanced by secondary metabolism (flavonoids and flavonols biosynthesis) and physical barrier construction (cuticle, suberin, and wax biosynthesis), consistent with the findings of Muhammad et al (Akhtar et al., 2026). The activation of cutin/suberin biosynthesis represents a strategic investment in root barrier formation. By synthesizing hydrophobic polymers in the endodermis, plants limit uncontrolled apoplastic flow of Na^+^ and water—an energy-efficient strategy that reduces the metabolic cost of ion exclusion at the plasma membrane level. A limitation of this study is that direct measurements of Na^+^ and K^+^ contents were not performed in this study. Although multiple lines of molecular and physiological evidence support the barrier-mediated restriction of Na^+^ influx, future work with direct ion content analysis is needed to fully validate this mechanism. Flavonoids are important antioxidants, and their enhanced synthesis corresponds to the early induction of antioxidant enzyme activity at the physiological level, jointly forming a chemical and enzymatic synergistic antioxidant defense system (Rao and Zheng, 2025). The activation of lipid barrier synthesis pathways such as cutin and suberin provides molecular evidence for restricting the uncontrolled flow of ions and water (Grünhofer et al., 2025), which, together with lignin accumulation in this study, points to the multi pathway synergy of root structure strengthening.

Under EHS stress, the sustained enrichment of phenylpropanoid biosynthesis is confirmed by physiological data of lignin precursor accumulation, revealing the importance of cell wall reinforcement in coping with severe osmotic stress (Pesquet et al., 2025). The disturbance of carbohydrate metabolism pathways such as galactose metabolism, starch and sucrose metabolism may reflect a profound restructuring of carbon source allocation, energy supply, and sugar signaling under EHS stress (Wang et al., 2013; Chen et al., 2026). Oxidized lipids derived from oxylipin/jasmonate-related metabolism, such as jasmonic acid precursors, can act as signaling molecules participating in stress response (Maynard et al., 2018; Resemann et al., 2023). Their activation under EHS stress interacts with lipid signaling and jasmonic acid signaling pathways triggered by intensified membrane lipid peroxidation, jointly regulating defense gene expression and programmed cell death (Lewis et al., 2023). Although the metabolites detected in this study were annotated as prostaglandin-like compounds based on database matching, plants lack the classical animal prostaglandin synthase pathway. These compounds are more likely to represent plant-specific oxylipins or jasmonate-related metabolites that participate in stress signaling through pathways involving lipoxygenases and allene oxide synthases. Their accumulation under salt stress may reflect activation of membrane lipid remodeling and oxidative signaling cascades rather than classical prostaglandin biosynthesis (Xia et al., 2026). Of course, future targeted metabolomics with authentic standards is needed to confirm their precise identities.

Transcriptional changes represent the immediate regulatory layer linking stress perception to physiological outputs. Transcriptommic analysis found that the number of DEGs increased with the severity of salt stress, indicating that *B. balsamifera* employs a survival strategy by mobilizing extensive genetic information in response to salt stress (Basharat et al., 2026). GO enrichment analysis indicates that the adaptive response of *B. balsamifera* under salt stress is driven by extensive transcription factor mediated transcriptional reprogramming. These transcription factors act as molecular switches, sensing and integrating upstream stress signals, thereby regulating the expression of downstream functional genes, including genes involved in cell wall modification, ion transport, osmoregulatory substance synthesis, and antioxidant defense (Aizaz et al., 2024; Admas et al., 2025). Concurrently, the enrichment of genes related to cell wall tissue directly supports the conclusion that roots adapt to stress through cell wall remodeling (lignification, suberin, etc.) at the transcriptional level. This finding is consistent with the results of Guo et al. (2026), suggesting that cell wall reinforcement represents a conserved adaptive strategy across different organs of this species under salt stress. Combined with KEGG analysis, it was found that Plant hormone signaling and MAPK signaling pathways were significantly enriched. This result is consistent with Wang et al.’s discussion on the synergistic effect of MAPK cascade as a conserved signaling module with hormone pathways (He et al., 2020), and also confirms Mahajan et al.’s view that ABA dependent transcription factors integrate stress signals (Mahajan et al., 2025). The sustained enrichment of the Phenylpropanoid biosynthesis pathway in the transcriptome, in concert with metabolomic and physiological data, forming a complete model of “gene up-regulation→enzyme activity/quantity increase→metabolic flow enhancement→final product (lignin) accumulation→physiological function (barrier formation)”, which is consistent with Rao and Zheng’s assertion on the core position of the phenylpropanoid pathway in stress adaptation (Rao and Zheng, 2025).

The combined analysis of metabolome and transcriptome strongly confirms the authenticity, importance, and synergy of multiple pathways in response to salt stress in *B. balsamifera* (Yu et al., 2025). From a functional perspective, the activation of these pathways reflects the multi-level salt stress adaptation strategy of *B. balsamifera* root. Firstly, by synthesizing hydrophobic lipid polymers (suberin/cutin) to enhance the selectivity of the root barrier, it constitutes a frontline physical strategy to restrict ion free diffusion and regulate water transport. Secondly, the activation of the Phenylpropanoid pathway simultaneously achieves dual functions of chemical defense (antioxidant flavonoids clearing ROS) and structural support (lignin reinforced cell wall). Finally, the oxylipin/jasmonate-related metabolism pathway is involved in membrane lipid-related metabolic processes and may contribute to oxidative stress responses by producing oxidized lipid derivatives. These strategies function as an integrated, multilayered defense network. The multi-omics analysis focused on HS and EHS to capture the molecular events associated with established and severe stress. It should be acknowledged that transcriptomic and metabolomic analyses were restricted to day 12, the time point at which most physiological indicators peaked. While this design captures the critical transition from stress adaptation to response, it does not resolve the full temporal dynamics of gene and metabolite regulation. In particular, day 6 may harbor early signaling events that warrant future investigation. Multi-time-point omics profiling would provide a more complete picture of the temporal sequence of salt responses in B. balsamifera roots.

It is worth mentioning that in this study conclusions regarding the “multilayered adaptive strategy” are derived primarily from correlative analyses, including co-expression patterns between transcripts and metabolites, and pathway co-enrichment from transcriptomics and metabolomics. While these analyses provide valuable systems-level insights, they do not establish direct causal relationships. Future studies employing genetic approaches—such as gene knockout or overexpression—and targeted metabolic flux analysis will be required to functionally validate the proposed roles of specific genes and metabolites in salt tolerance.

## Conclusion

This study systematically revealed the multi-level adaptation mechanism of the characteristic medicinal plant *B. balsamifera* in response to salt stress by integrating phenotype observation, physiological and biochemical changes, as well as transcriptome and metabolomics. The research has shown that salt stress inhibits its growth, leading to leaf damage and reduced biomass. The roots responded to stress by dynamically regulating antioxidant enzyme activity, accumulating osmoregulatory substances, and enhancing lignification. However, EHS stress exceeded its physiological regulatory threshold, resulting in decreased antioxidant enzyme activity and exacerbated membrane lipid peroxidation. Transcriptomic analysis revealed large-scale transcriptional reprogramming, with DEGs enriched in transcriptional regulation, cell wall tissue, and hormone signaling pathways. Metabolomics indicated that under HS stress, secondary metabolism (such as flavonoid synthesis) and physical barrier construction (such as cutin/suberin synthesis) pathways were mainly activated. Under EHS stress, the focus was on cell wall strengthening (phenylpropanoid pathway), energy metabolism restructuring (Starch and sucrose metabolism), and stress signal transduction (oxylipin/jasmonate-related metabolism). Multi-omics integration confirmed that cutin/suberin synthesis, phenylpropanoid biosynthesis, and oxylipin/jasmonate-related metabolism are core synergistic response pathways, corresponding to physical barrier construction, chemical defense and structural support, and membrane lipid signaling regulation functions, respectively. It can be seen that *B. balsamifera* responds to salt stress by integrating a multi-level network of morphology-physiology-transcription-metabolism, and its salt tolerance is associated with the synergistic balance among antioxidant defense, osmotic regulation, transcriptional control, and structural barriers. This study provides new insights into the salt adaptation mechanism of *B. balsamifera* and offers a theoretical basis for the breeding of new salt-tolerant varieties and the regulation of cultivation practices.

## Materials and methods

### Plant materials and salt stress treatment

In April 2024, the *B. balsamifera* seeds were collected from Duanqiao Town, Guanling County, Anshun City, Guizhou Province, China (N 19°30′36″, E 109°34′12″). The materials originated from the nursery’s cultivated germplasm bank, which has been domesticated and preserved long-term, belonging to artificially propagated non-wild populations. The seeds were obtained with the permission of Guizhou Qinyaoyuan Zhongxin Pharmaceutical Investment Development Co., Ltd., the managing company of the germplasm bank, and the collection was authorized by the company’s general manager, Mr. Shi Yongyong. As the material was sourced from a managed cultivation facility rather than from wild populations, no specific permits for wild plant collection were required. The seeds were then germinated and cultivated in greenhouse No.2 of the Medicinal Plant Cultivation Experimental Field at the same university (N 26°22′20″, E 106°27′34″). In June 2024, five-month-old seedlings with uniform growth (mean height: 11.67 cm) were selected for subsequent salt stress experiments. We ensured that the collection of plant material and the conduct of experimental research complied with relevant institutional, national, and international guidelines and legislation.

Based on preliminary experimental results, in which plants treated with 100 mmol·L^−^¹ NaCl remained healthy with no visible mortality within two weeks, five NaCl concentration gradients were established: 0 mmol·L^−^¹ (control, CK), 50 mmol·L^−^¹ (low salt, LS), 100 mmol·L^−^¹ (medium salt, MS), 150 mmol·L^−^¹ (high salt, HS), and 200 mmol·L^−^¹ (extremely high salt, EHS). The experimental unit was one pot (one plant per pot), with three independent pots per treatment per time point (n = 3). At each sampling time point, independent plants were destructively harvested for root tissue collection, and thus data from different time points represent independent biological samples. During the experimental period, 100 mL of the corresponding NaCl solution was applied to each pot between 7:00 and 8:00 AM every three days, ensuring that excess solution drained from the bottom to maintain uniform salt concentration in the growth substrate. On sampling days (days 6, 12, 18, and 24), samples were collected prior to the scheduled irrigation; on non-sampling days, irrigation was performed according to the same schedule.

Sampling was conducted at 0, 6, 12, 18, and 24 days after the initiation of treatment. For each treatment, three plants were randomly selected. Whole root samples were collected as follows: root-associated debris was removed, and roots were thoroughly rinsed with sterile water and blotted dry. The entire root system was excised at the base using sterilized scissors, then chopped and homogenized before being aliquoted into sterile cryogenic vials. Samples were immediately flash-frozen in liquid nitrogen and stored at −80 °C for subsequent analysis.

### Measurement of physiological parameters

All physiological indices were determined using the microplate method with commercial kits produced by Grace Biotechnology Co., Ltd. (Suzhou, China). The specific measurement methods are as follows: root antioxidant parameters—including malondialdehyde (MDA) content, superoxide dismutase (SOD), catalase (CAT), and peroxidase (POD) activities—were assessed using the thiobarbituric acid colorimetric method, WST-8 method, ultraviolet spectrophotometry, and guaiacol colorimetric method, respectively. Osmoregulatory indicators, namely lignin (LIG), soluble sugar (SS), soluble protein (SP), and proline (PRO) contents, were quantified by acetylation, anthrone colorimetric, Coomassie brilliant blue, and ninhydrin colorimetric methods, respectively.

### Determination of key sampling time points for metabolome and transcriptome analyses

Based on the time-course physiological data from *B. balsamifera* roots, significant differences in most physiological parameters under LS and MS treatments relative to CK were not evident until 12th or even 18th day. By contrast, under HS and EHS treatments, significant deviations from CK were detectable as early as 6th day. Notably, key indicators such as MDA, CAT, POD, SOD, and SP reached their peak levels on the 12th day, indicating that the plant had entered a critical transition stage from stress adaptation to salt response at this time point. Therefore, samples from CK, HS, and EHS on the 12th day were selected for metabolomic and transcriptomic sequencing analysis, aiming to reveal the differential expression and regulatory mechanisms of key genes in response to salt stress in *B. balsamifera*.

### Metabolomics sequencing analysis

Metabolomic profiling was performed by Shanghai Majorbio Biotechnology Co., Ltd. (Shanghai, China) following the company’s standard protocols. Ultra-high performance liquid chromatography tandem Fourier transform mass spectrometry (UHPLC-Q Exactive HF-X, Thermo Fisher Scientific, USA) coupled with an HSS T3 column (100 mm × 2.1 mm i.d., 1.8 µm, Waters, USA) was used. The extraction and derivatization of metabolites followed the method of Han et al. (2024). Chromatographic separation was performed using a gradient elution program (Ren et al., 2022). The raw data underwent baseline filtering, peak detection, integration, and retention time correction using Progenesis QI (Waters Corporation, Milford, USA). Metabolite information was obtained by matching against the HMDB (http://www.hmdb.ca/), Metlin (https://metlin.scripps.edu/), and the in-house library of Shanghai Majorbio Biotechnology Co., Ltd. The data matrix was processed by removing missing values using the 80% rule, fill in minimum values, performing sum normalization, excluding variables with QC sample RSD > 30%, and applying log10 transformation.

Differential metabolites were identified by combining univariate and multivariate statistical analyses. For univariate analysis, fold change (FC) and an unpaired two-tailed Student’s *t*-test were used to assess the magnitude and significance of differences between groups. For multivariate analysis, orthogonal partial least squares discriminant analysis (OPLS-DA) was performed using the ropls package (Version 1.6.2) in R with 7-fold cross-validation. Model reliability and goodness-of-fit were evaluated by a permutation test with 200 permutations, and the R²X, R²Y, and Q² values were calculated to assess model performance. For both HS vs CK and EHS vs CK comparisons, the Q² values were well above 0.5 (0.957 and 0.982, respectively), and the small gap between R²Y and Q² indicated that the models were not overfitted (**Supplementary Table S1**). The variable importance in projection (VIP) value of each metabolite was then obtained from the validated OPLS-DA model to evaluate its contribution to group separation. Metabolites satisfying fold change ≥ 1.5, VIP ≥ 1, and FDR-adjusted p < 0.05 (Benjamini-Hochberg correction) were considered differentially accumulated metabolites (DAMs) (Liu et al., 2023). Differential metabolites were annotated using the KEGG database to obtain the biological pathways they are involved in (https://www.kegg.jp/kegg/pathway.html).

### RNA sequencing and data analysis

RNA sequencing was performed by Shanghai Majorbio Biotechnology Co., Ltd. (Shanghai, China) following the company’s standard protocols. Total RNA was extracted from each of the nine samples using the RNAprep Pure Plant Total RNA Extraction Kit. The concentration and purity of the isolated RNA were measured with a Nanodrop2000 spectrophotometer. RNA integrity was evaluated by agarose gel electrophoresis, and RNA integrity number (RIN) values were determined on an Agilent 2100 Bioanalyzer. The sequencing was performed on an Illumina HiSeqXT0n platform by Shanghai Meiji Biotechnology Co., Ltd. Since *B. balsamifera* lacks a reference genome, after obtaining high-quality RNA-seq sequencing data, Trinity software was used to generate contigs and singletons from all quality-controlled sequencing reads through *de novo* assembly. Subsequently, TransRate (http://hibberdlab.com/transrate/) and CD-HIT (http://weizhongli-lab.org/cd-hit/) were employed to filter and optimize the sequences obtained from the *de novo* assembly of the transcriptome. Finally, BUSCO (Benchmarking Universal Single-Copy Orthologs, http://busco.ezlab.org) was used to evaluate the assembly completeness of the transcriptome (Chen et al., 2018). The screening criteria included: removing read lengths containing adapter sequences, read lengths with unknown base (N) ratios exceeding 5%, and read lengths with base ratios exceeding 20% for quality scores ≤ 20. The high-quality and clean data obtained were used for subsequent analysis. Sample correlation analysis was performed to assess the reproducibility of biological replicates. Within-group correlations were high (R² > 0.99) for all treatment groups, while inter-group correlations were lower, consistent with the strong treatment effects. No samples were identified as outliers, and all samples were retained for downstream analysis. Differential gene expression was analyzed using DESeq2 (Oh et al., 2023). P-values were adjusted for multiple testing using the Benjamini-Hochberg method to control the false discovery rate. Genes with an adjusted P-value (FDR) < 0.05 and |log2 fold change| ≥ 0.585 were considered differentially expressed. Functional annotation of DEGs was conducted via the GO and KEGG databases. Enrichment analysis was then performed using an R-based tool to associate these genes with specific GO categories and KEGG pathways, elucidating their biological functions (Conesa et al., 2005; Mao and T.C.J.G., 2005).

### Correlation analysis between metabolome and transcriptome

To investigate the transcriptional regulatory mechanisms associated with salt-tolerant metabolic pathways in *B. balsamifera*, Procrustes analysis was conducted to examine relationships between metabolomic and transcriptomic datasets. We employed Principal Component Analysis (PCA) to ordinate the transcriptomic and metabolomic data separately, and then performed Procrustes superposition to evaluate the concordance of sample distribution patterns across different treatment groups (Greenacre et al., 2021). For integrated metabolomic and transcriptomic analysis, all data were log2-transformed prior to correlation analysis. Pearson correlation coefficients were calculated between all DEGs and DAMs in the HS vs CK and EHS vs CK comparison groups. Significant gene–metabolite pairs were retained based on |ρ| > 0.80 and *P* ≤ 0.05 (Li et al., 2021). To correct for multiple testing, the Benjamini–Hochberg false discovery rate (FDR) procedure was applied, and adjusted *P* ≤ 0.05 was considered statistically significant. All the above analysis were performed using the Majorbio Cloud platform. To clarify the key KEGG pathways in response to salt stress in *B. balsamifera* root, the focus is on analyzing the important pathways co enriched in transcriptome and metabolome analysis, as well as their associated genes and metabolites.

### Determination of gene expression levels (RT-qPCR)

This study selected nine genes related to salt stress response for qRT-PCR validation. Fluorescent primers for these genes were designed using NCBI’s online primer design tools and synthesized by Beijing Tsingke Biotech Co., Ltd. The detailed information of these genes was listed in **Supplementary Table S2**. Total RNA was isolated using the RNAprep Pure Plant RNA Mini Kit (TIANGEN Biotechnology Co., Ltd.), and reverse transcription into cDNA was performed using the FastKing gDNA Dispelling RT SuperMix from the same supplier. All procedures were performed in accordance with the manufacturer’s specifications. The *18S rRNA* gene served as a reference for normalization and RT-qPCR was performed to measure the expression level of nine genes. All qPCR reactions were performed using the SYBR PRIME qPCR Kit (Fast HS; Baoguang Biological, Cat. No. BG0014) as directed by the manufacturer. Amplification was performed on an Applied Biosystems 7500 Real-Time PCR System (Life Technologies, Beverly, MA, USA). Relative expression levels were calculated using the 2 ^-ΔΔCt^ method (Arocho et al., 2006).

### Data statistics and analysis

SPSS 22.0 and Excel 2021 were used for data statistical analysis, and GraphPad Prism 9.5 and OriginPro 2024 were used for plotting. For each sampling time point, one-way ANOVA followed by Duncan’s multiple range test was used to compare the effects of different salt treatments (CK, LS, MS, HS, EHS) on each parameter. This time-point-wise analysis was chosen because the primary aim was to assess treatment differences at each stage of stress progression. All data are presented as mean ± standard deviation (n = 3). Differences were considered significant at P < 0.05. The significant differences between different treatments at the same treatment time were marked with different lowercase letters (*P* < 0.05).

## Data Availability

The raw transcriptome sequencing data have been deposited in the NCBI public repository. Details regarding the database and accession number are provided below: https://www.ncbi.nlm.nih.gov/sra/SRP649295.
