# Supplementary material for: Unveiling the adaptive strategies in Blumea Balsamifera roots under salt stress through integrated physiological and multi-omics analysis
Source: Front Plant Sci. 2026 Oct 7;17:1970547. doi: 10.3389/fpls.2026.1970547 (PMC13683093; doi:10.3389/fpls.2026.1970547)
Supplement: Supplementary file 1 [file Table1.docx]

**Table S1 OPLS-DA model parameters for HS vs CK and EHS vs CK comparisons.**

| **Comparison** | **Component** | **R²X** | **R²X (cum)** | **R²Y** | **R²Y (cum)** | **Q²** | **Q² (cum)** |
| --- | --- | --- | --- | --- | --- | --- | --- |
| HS vs CK | p1 | 0.381 | 0.381 | 0.958 | 0.958 | 0.909 | 0.909 |
| HS vs CK | o1 | 0.197 | 0.578 | 0.0323 | 0.0323 | 0.0488 | 0.0488 |
| HS vs CK | sum | — | 0.578 | — | 0.991 | — | 0.957 |
| EHS vs CK | p1 | 0.476 | 0.476 | 0.978 | 0.978 | 0.957 | 0.957 |
| EHS vs CK | o1 | 0.284 | 0.760 | 0.0175 | 0.0175 | 0.0245 | 0.0245 |
| EHS vs CK | sum | — | 0.760 | — | 0.996 | — | 0.982 |

Note: p1, principal component; o1, first orthogonal component; cum, cumulative. Q² > 0.5 indicates good predictive ability.
