# Supplementary material for: Unveiling the adaptive strategies in Blumea Balsamifera roots under salt stress through integrated physiological and multi-omics analysis
Source: Front Plant Sci. 2026 Oct 7;17:1970547. doi: 10.3389/fpls.2026.1970547 (PMC13683093; doi:10.3389/fpls.2026.1970547)
Supplement: Supplementary file 2 [file Table2.docx]

| **Table S2 Primers and gene annotations for RT-qPCR validation of salt-responsive genes** | | | | | |
| --- | --- | --- | --- | --- | --- |
| **Gene ID** | **Gene name** | **Primer Name** | **Primer Sequence** | **Product Size** | **Predicted Function** |
| TRINITY_DN12832_c0_g1 | ERF | DN12832_F | CCAGCCGCCTTCACTCAA | 93 | ethylene-activated signaling pathway |
|  |  | DN12832_R | CGTCGAAGCCGGTGGTTA | 93 |  |
| TRINITY_DN2910_c0_g2 | LRR receptor kinase BAK1-like | DN2910_F | TGCACACAAGCCACACCA | 89 | protein serine/threonine kinase activity |
|  |  | DN2910_R | TCGTCCCATCTCTCCGCT | 89 |  |
| TRINITY_DN5978_c0_g1 | CDPK-related kinase 5-like | DN5978_F | AGCTGTGGTGGGTCTGGA | 84 | calmodulin-dependent protein kinase activity |
|  |  | DN5978_R | CCACGCCCCAATTCGTCT | 84 |  |
| TRINITY_DN2822_c0_g2 | phenylalanine ammonia lyase | DN2822_F | ACCAAGCTCCTCAACGCC | 80 | L-phenylalanine catabolic process |
|  |  | DN2822_R | AGTGGAACAAGGTCGCCG | 80 |  |
| TRINITY_DN32008_c0_g3 | peroxidase 49 | DN32008_F | CCAGTGACCATCTCCGCC | 83 | response to oxidative stress |
|  |  | DN32008_R | CGGACGATGAGCTCTGCA | 83 |  |
| TRINITY_DN5542_c0_g1 | peroxidase 5 isoform X2 | DN5542_F | GTGACCCCGTGACCAGTC | 93 | hydrogen peroxide catabolic process |
|  |  | DN5542_R | TCCCCATCTTGACCATGGC | 93 |  |
| TRINITY_DN9442_c0_g3 | peroxidase 2 | DN9442_F | TGTAGAAGCCGCATGCCC | 111 | response to oxidative stress |
|  |  | DN9442_R | CTTCCCAAGGGCACTGGC | 111 |  |
| TRINITY_DN5087_c0_g1 | peroxidase P7 | DN5087_F | ACCGGTGAAAAGAGGGCG | 110 | response to oxidative stress |
|  |  | DN5087_R | GCACACGAGACCACACCA | 110 |  |
| TRINITY_DN10051_c0_g1 | 60S acidic ribosomal protein P1-like | DN10051_F | ATGTTGGTGCTGGTGGCG | 87 | protein kinase activator activity |
|  |  | DN10051_R | CAGGAGGAGCAGCTGCAG | 87 |  |
| 18S | reference gene | 18S_F | CGGCTACCACATCCAAGGAA | 185 | Purpose: Correct experimental errors |
|  |  | 18S_R | GCTGGAATTACCGCGGCT | 185 |  |
